# Comparative proteomic analysis of children FSGS FFPE tissues

**DOI:** 10.1186/s12887-022-03764-7

**Published:** 2022-12-12

**Authors:** Jiajia Ni, Sha Tian, Lin Bai, Qianying Lv, Jialu Liu, Jiaojiao Liu, Ye Fang, Yihui Zhai, Qian Shen, Jia Rao, Chen Ding, Hong Xu

**Affiliations:** 1 Department of Nephrology, Children’s Hospital of Fudan University, National Pediatric Medical Center of China, Shanghai, China; 2 Kidney Development and Pediatric Kidney Disease Research Center, Shanghai, China; 3 State Key Laboratory of Genetic Engineering and Collaborative Innovation Center for Genetics and Development, School of Life Sciences, Institute of Biomedical Sciences, Human Phenome Institute, Zhongshan Hospital, Fudan University, Shanghai, 200433 China

**Keywords:** Focal segmental glomerulosclerosis, Steroid resistance, Proteomics

## Abstract

**Background:**

In children, focal segmental glomerulosclerosis (FSGS) is the main cause of steroid resistant nephrotic syndrome (SRNS). To identify specific candidates and the mechanism of steroid resistance, we examined the formalin-fixed paraffin embedded (FFPE) renal tissue protein profiles via liquid chromatography tandem mass spectrometry (LC-MS/MS).

**Methods:**

Renal biopsies from seven steroid-sensitive (SS) and eleven steroid-resistant (SR) children FSGS patients were obtained. We examined the formalin-fixed paraffin embedded (FFPE) renal tissue protein profiles via liquid chromatography tandem mass spectrometry (LC-MS/MS). Kyoto Encyclopedia of Genes and Genomes (KEGG) enrichment and Gene Ontology (GO) analysis, as well as the construction of protein-protein interaction (PPI) network were performed. Two proteins were further valiadated by immunohistochemistry staining in FSGS patients and mice models.

**Results:**

In total, we quantified more than 4000 proteins, of which 325 were found to be differentially expressed proteins (DEPs) between the SS and SR group (foldchange ≥2, *P*<0.05). The results of GO revealed that the most significant up-regulated proteins were primarily related to protein transportation, regulation of the complement activation process and cytolysis. Moreover, clustering analysis showed differences in the pathways (lysosome, terminal pathway of complement) between the two groups. Among these potential candidates, validation analyses for LAMP1 and ACSL4 were conducted. LAMP1 was observed to have a higher expression in glomerulus, while ACSL4 was expressed more in tubular epithelial cells.

**Conclusions:**

In this study, the potential mechanism and candidates related to steroid resistance in children FSGS patients were identified. It could be helpful in identifying potential therapeutic targets and predicting outcomes with these proteomic changes for children FSGS patients.

**Supplementary Information:**

The online version contains supplementary material available at 10.1186/s12887-022-03764-7.

## Introduction

Focal segmental glomerulosclerosis (FSGS) is a group of clinicopathological syndromes sharing a common glomerular lesion [1]. FSGS patients, however, are likely to do poorly on glucocorticoids and progress to end-stage renal disease (ESRD). The 2012 KDIGO guidelines recommend that calcineurin inhibitors (CNIs) are the first choice for children with steroid-resistant nephrotic syndrome, excluding inherited nephrotic syndrome [2]. Nevertheless, CNIs can be costly, and they can cause severe side effects, such as nephrotoxicity and infections. Therefore, identifying the underlying mechanism and candidates for FSGS steroid resistance is urgently required.

Proteomics has been widely applied to investigate the mechanism underlying diseases and to identify biomarkers for the diagnosis and prognosis of various diseases. Based on MS/MS sequencing, the LC-MS/MS platform is outstanding for protein identification, even with only one peptide [3]. Previous proteomics studies on various glomerular diseases have made substantial efforts to identify candidates in urine or serum, thus predicting prognosis and avoiding invasive renal biopsy, which can cause complications including haematoma, infection and arteriovenous fistula [4, 5]. There have been several biomarkers suggested to help diagnose FSGS-SR and FSGS-SS or minimal change disease (MCD) and FSGS, but none of them are clinically available to date [6–8]. Despite the convenience of collecting urine or blood samples, the complex protein components in blood or urine and the large dynamic range of changes make the identification and quantification of proteins particularly complicated [9, 10]. However, abnormal proteins appear earlier in tissue samples, and the local concentrations are higher. FFPE blocks of kidney tissue are the most common specimens for research. Recent advances in FFPE protein extraction combined with tandem mass spectrometry made it possible to quantify proteins in stored biopsies at a large scale [11, 12]. The large amount of stored FFPE kidney tissues presents substantial opportunities for investigating the proteomic basis of renal disease.

Here we applied FFPE proteomics using LC-MS/MS to identify the mechanism and candidates related to steroid resistance in children FSGS patients. Further examination of two of the significantly up-regulated proteins was carried out in the kidneys of FSGS patients and mice models.

## Materials and methods

### Patients

This study was approved by the institutional review board of the Children’s Hospital of Fudan University and was conducted according to the principles of the Helsinki Declaration. In total, 18 patients with biopsy proven FSGS (seven with steroid sensitivity and eleven with steroid resistance) were enrolled. It was defined as FSGS-SS when urine remained negative after 4–6 weeks of steroid treatment. The FSGS-SR group exhibited steroid resistance after 6 weeks of treatment and whose urinary protein level continuously exceeded +++ (above 50 mg/kg.d). As controls in the validation stage, paracarcinoma kidney tissue was obtained from patients who underwent renal carcinoma resection.

### Deparaffinization and sample preparation

FFPE biobank specimens (10 μm thick) were first deparaffinized by two washes in xylene (5 min at 37 °C each), followed by washes in absolute ethanol, 90% ethanol, 85% ethanol and 75% ethanol. The sections were air-dried and incubated in nearly 50 μL TCEP buffer (2% deoxycholic acid sodium salt, 40 mM 2-chloroacetamide, 100 mM tris-phosphine hydrochloride, 10 mM (2-carboxyl)-phosphine hydrochloride, 1 mM phenylmethylsulfonyl fluoride mixed with MS water, pH 8.5), heated at 99 °C for 30 minutes and cooled to room temperature. Then, trypsin (Promega) was used to digest the samples overnight at 37 °C. After adding 13 μL of 10% formic acid to each tube, vortexing was performed for 3 min, followed by a sedimentation period of 5 min (12,000 g). To extract the supernatant, a new 1.5-mL tube with 350 μL buffer (0.1% formic acid in 50% acetonitrile) was used (vortex for 3 min and then sediment at 12,000 g for 5 min). Then, a new tube was used to dry the supernatant in a 60 °C vacuum drier. After drying, 100 μL of 0.1% formic acid was added to dissolve the peptides, which were vortexed for 3 min and then sedimented for 3 min (12,000 g). To prepare for desalination, the activation of pillars with 2 slides of 3 M C18 disk was required, and the lipid was loaded as follows: 90 μL 100% acetonitrile twice, 90 μL 50 and 80% acetonitrile once in turn, and then 90 μL 50% acetonitrile once. After pillar balance with 90 μL 0.1% formic acid twice, the supernatant of the tubes was loaded into the pillar twice, and decontaminated with 90 μL 0.1% formic acid twice. Finally, 90 μL elution buffer (0.1% formic acid in 50% acetonitrile) was added to the pillar fir elution twice and only the effluent was collected for MS before being dried with a vacuum concentrator (Thermo Scientific). The sample preparation was conducted as previously described [13, 14].

### Liquid chromatography tandem mass spectrometry

Samples were suspended in an appropriate buffer and analysed on a Q Exactive HF-X mass Spectrometer (Thermo Fisher Scientific, Rockford, IL, USA) coupled with a high-performance liquid chromatography system (EASY nLC 1200, Thermo Fisher). Redissolved dried peptide samples were loaded onto the 150 μm by 2 cm ReproSil-Pur C18-AQ column (3 μm; Dr. Maisch) in Solvent A (0.1% formic acid in water), with a maximum pressure of 280 bar using Solvent A. Separation was then performed on a home-made 100 μm by 15 cm silica microcolumn using mobile phase B with a gradient of 4–100% (0.1% Formic acid in 80% ACN) at a flow rate of 600 nl/min for 75 min. Mass spectrometry was conducted under a data-dependent acquisition mode after the elution of peptides. The orbitrap instrument was used to conduct the MS1 full scan by scanning 300–1400 m/z at120,000 resolution. The maximal ion injection time was 80 ms with an automatic gain control (AGC) of 3e6. A top-speed MS2 acquisition was performed and selected precursor ions were subjected to higher energy collision dissociation (HCD) with 27% normalized collision energy. AGC at 5e4 was applied to analyze fragment ions. A maximum ion injection time was achieved by MS2 of 20 ms, while and the dynamic exclusion was 12 s. Data acquisition was performed with Xcalibur software (Thermo Scientific).

### Protein identification

Maxquant (version 1.5.3.30) was used to search raw files against the human Refseq protein database (updated on 04-07-2013, 32,015 entries) of National Center for Biotechnology Information, using the integrated Andromeda search engine with the false discovery rate (FDR) < 1% at peptide and protein levels [15]. 20 ppm mass tolerances were set for the precursor and a 0.5 Da was set for productions on the Fusion Lumos. K and R were proteolytic cleavage sites. A maximum of two missed cleavages was allowed. Carbamidomethyl (C) was considered as a fixed modification. Variable modifications included N-acetylation and oxidation of methionine. MaxQuant was used to quantify all identified peptides based on their MS1 intensities. Peptide FDR was adjusted to 1%. For protein quantifications, an intensity-based absolute quantification (iBAQ) approach was used, which divided the protein abundance (derived from identified peptides’ intensities) by the number of theoretically observable peptides as previously described [16]. By using a match between runs, it was possible to transfer the identification between LC-MS/MS runs based on their mass accuracy and retention time. Normalization to fraction of total (FOT) was utilized. iFOT is equal to the iBAQ of each protein divided by the sum of iBAQ of all proteins in the sample (iFOT = iBAQ/∑iBAQ * 10^5^) [17].

### Protein GO term and KEGG pathway enrichment analyses

The DAVID open-source program, version 6.7, was applied to perform gene ontology (cellular components, biological processes and molecular functions) and KEGG pathway enrichment analyses (http://david.abcc.ncifcrf.gov/home.jsp) [18, 19].

### Protein-protein interaction network analyses

The STRING database was used to conduct protein-protein interaction (PPI) network analyses. Cyto-Hubba was applied to discover the hub genes in the PPI network of differential proteins [20]. In this study, we mined the top 10 hub genes by that method.

### Lipopolysaccharide (LPS) mice model

Animals used in the LPS study were C57BL/6 mice kept in the Fudan university and experiments were approved by the Animal Care and Use Committee. Saline and LPS (Sigma) were both administrated to each group of six mice once per day for 3 days. The LPS group with 10 mg per kg for 3 days via intraperitoneal injection to develop the nephropathy model. Experiments were performed and reported in accordance with the ARRIVE guidelines.

### Immunohistochemistry staining

kidney tissues from human and mouse were obtained and immunohistochemical staining was performed. Paraffin-embedded sections were deparaffinized and hydrated using xylene and a graded series of ethanol concentrations. Then sections were heated to 100 °C for 20 min in citrate buffer (pH 6.0) and blocked with 3% hydrogen peroxide. After blocking with 10% goat serum in PBS for an hour at room temperature, tissues were stained with an anti-LAMP-1 antibody (Abcam, ab25630; dilution 1:25) and anti-ACSL4 antibody (Abcam, ab155282; dilution 1:250). Afterwards, secondary antibody was added. Visualization was performed by diaminoben-zidine tetrahydrochloride (DAB). After counter-staining with Mayer’s hematoxylin (Sigma-Aldrich), all sections were evaluated under a 40-x objective light microscope.

### Statistical analysis

All data are presented as the mean ± standard error of the mean. Data between two groups were compared with the unpaired t test. Values of *P < 0.05 were considered significant in all analyses.

## Results

### Strategy for profiling proteins associated with FSGS steroid resistance in FFPE tissue

**Fig. 1:**
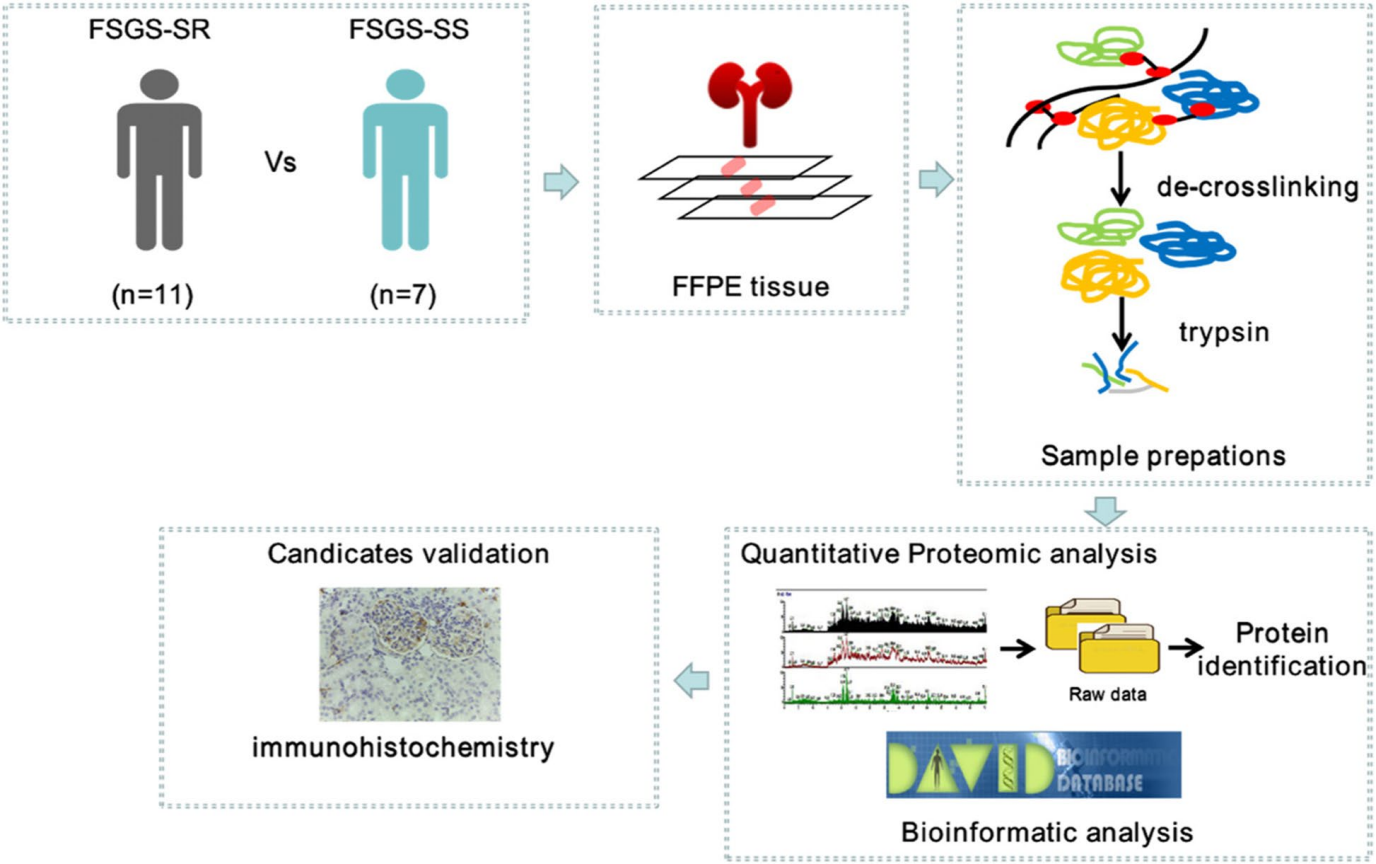
Overview of the study design. The human FFPE kidney tissue samples were subjected to proteomics analysis, and proteins were identified. Subsequently, DAVID online software was used to analyse the identified differentially expressed proteins. Finally, different laboratory methods were used to confirm the identified proteins

In order to discover candicates associated with FSGS progression and steroid resistance in FFPE tissue, a workflow was developed. An overview of the overall multistep workflow was depicted in Fig. 1.

### Clinical and laboratory characteristics of the patients

As shown in Table 1, clinical and laboratory information for the patients was provided. We enrolled eighteen patients with biopsy proven FSGS (sixteen males and two females). A single pathologist reviewed the biopsy samples. There was no evidence of secondary FSGS. No significant difference was found between the two groups of patients regarding their baseline clinical parameters.

**Table 1:** Clinical characteristics of the FSGS patients

	FSGS-SR	FSGS-SS	*P* value
Age (y)	5.94 ± 1.167 (range, 1.25–12)	4.344 ± 1.017 (range, 0.75–9)	0.356
Girl/boy	2/9	0/7	/
Weight (kg)	36.87 ± 5.559	23.64 ± 6.008	0.138
Height (cm)	132.8 ± 7.963	109.6 ± 11.85	0.110
Serum albumin (g/L)	21.99 ± 3.098	24.67 ± 4.183	0.607
Serum cholesterol (μmol/L)	9.332 ± 1.17	9.214 ± 2.51	0.963
Serum urea nitrogen (mg/dL)	7.964 ± 1.423	4.357 ± 0.709	0.074
Serum creatinine (μmol/L)	45.45 ± 11.41	30.43 ± 3.854	0.324
24 h Urine protein (g/d)	10.63 ± 3.345	3.417 ± 1.446	0.12

### Protein identification

**Fig. 2:**
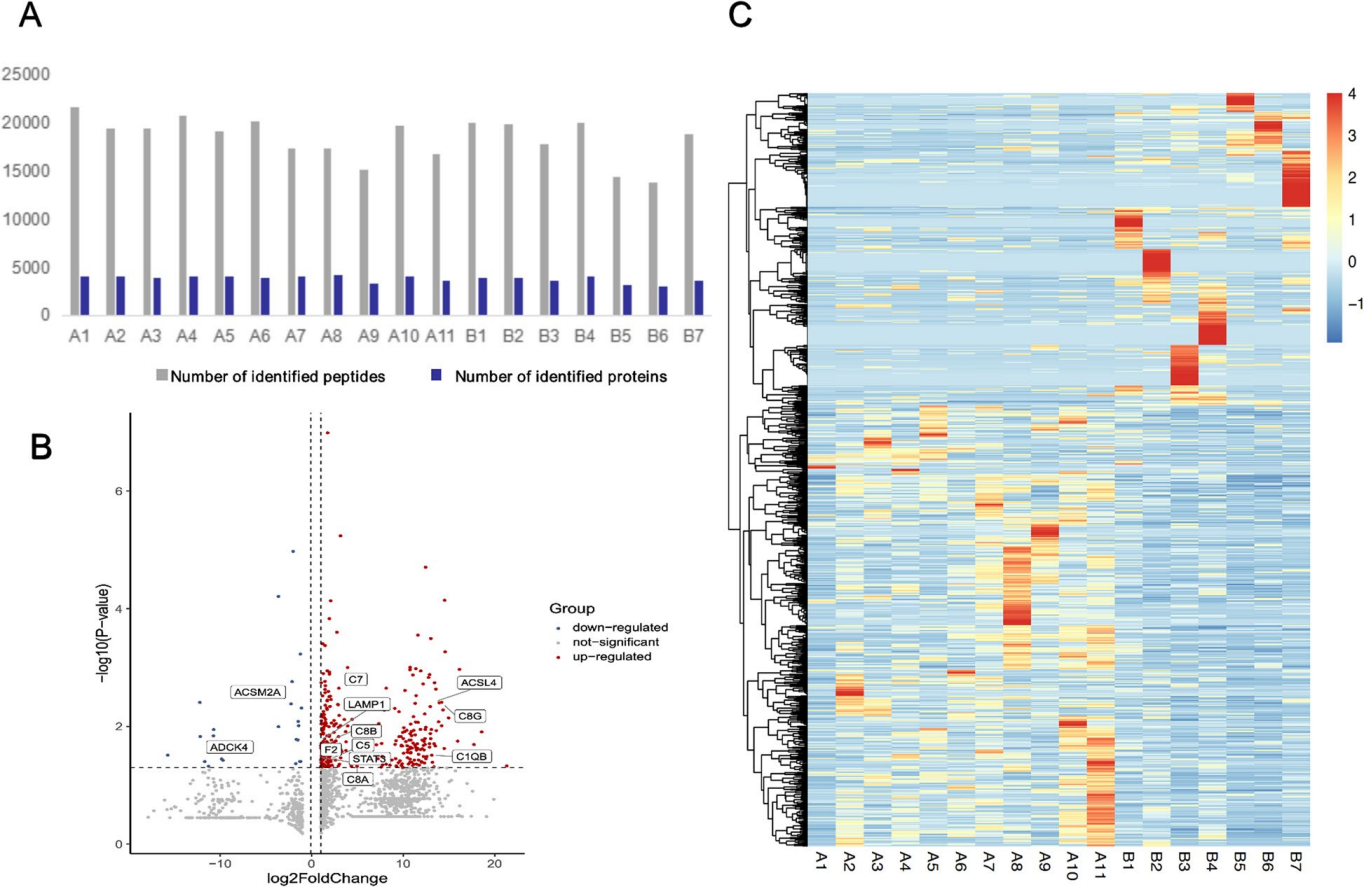
Differential expression (DE) analysis of quantitative proteomic profiling data. **A** The identified peptides (grey) and the quantified proteins (blue) in each sample were visualized as bar charts. **B** Volcano plot of DE proteins, a total of 302 proteins were up-regulated, a total of 23 proteins were down-regulated. Red dots stood for upregulated proteins, blue dots stood for downregulated proteins and grey indicated no significant difference. **C** Proteins differentially expressed between FSGS-SR and FSGS-SS samples, depicted as a heatmap and hierarchical clustering pattern. A1-A11 indicated FSGS-SR patients. B1-B7 indicated FSGS-SS patients

Each sample had a protein number ranging from 3131 to 4233 (Fig. 2A). A normalization procedure was implemented to remove systematic bias across comparison groups and total 1795 differentially expressed protein groups (DEPs) were identified (supplementary file 1). The data set after the reprocessing (fold change ≥2, *P* < 0.05) procedure was reduced to 302 up-regulated proteins and 23 down-regulated proteins in FSGS-SR, as shown in Fig. 2 B (volcano plot). Among the up-regulated proteins, there were several known glomerular disease associated proteins, such as the transcription factors signal transducer and activator of transcription 3, complement compoents [21, 22]. In contrast, the expression levels of proteins such as the typical kinase coenzyme Q8B, and acyl-coenzyme A synthetase (ACSM2A) were significantly down-regulated.

### GO term enrichment analysis

**Fig. 3:**
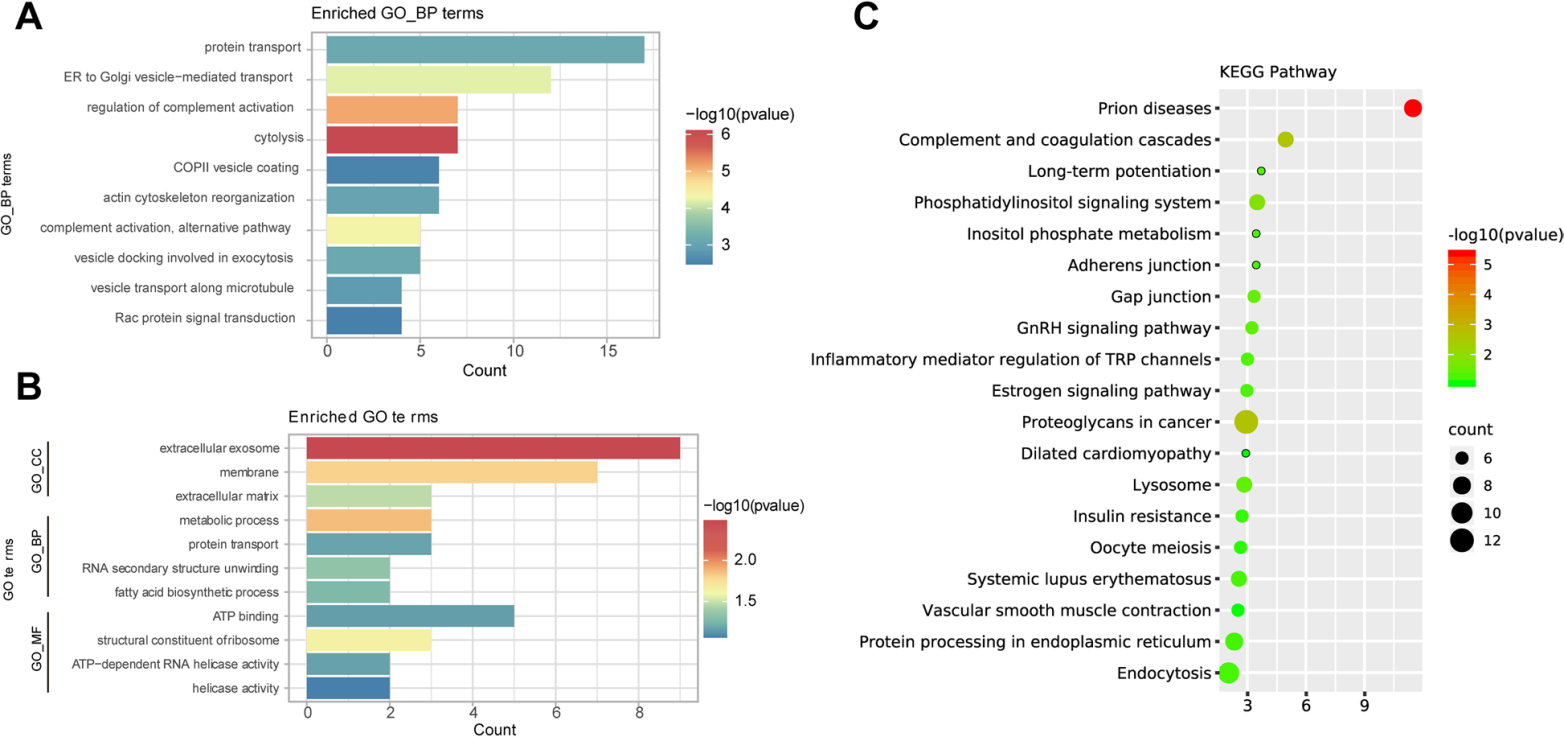
Enrichment GO function analysis of the upregulated and downregulated proteins with significant differences between the groups. Only the leading terms of each group are presented. **A** GOBP terms analysis of upregulated proteins. **B** GO analysis of downregulated proteins. **C** KEGG pathway analysis. GOCC, gene ontology term for cellular compoents; GOBP, gene ontology term for biological process; GOMF, gene ontology term for molecular function; KEGG, Kyoto Encyclopedia of Genes and Genomes

GO enrichment using DAVID software characterized the DEPs in the cell component (CC), biological process (BP), and molecular function (MF) categories. Each of the top 10 categories was calculated based on the protein counts, and the results are shown in Fig. 3A and B and supplementary Fig. 1A, B. Most of the up-regulated proteins were located in the cytosol, extracellular exosome and membrane (supplementary Fig. 1A). For the analysis of BP, the majority of the obtained proteins were shown to be involved in protein transport, or regulation of the complement activation process, cytolysis and actin cytoskeleton reorganization (Fig. 3A). In terms of MF, the results indicated that protein binding and Rac GTPase binding are mainly important functions (supplementary Fig. 1B). Among the down-regulated proteins, extracellular exosomes, metabolic processes and regulation of ATP binding were significantly enriched (Fig. 3B).

### KEGG pathway analysis of DEPs

KEGG enrichment highlighted 19 accumulated pathways involving the up-regulated proteins (URPs) (Fig. 3C). Interestingly, seven URPs (*p*-value = 0.0026) accumulated in the pathway of complement, and seven UPRs (*p*-value = 0.036) accumulated in the lysosome pathway. KEGG pathways in detail was shown in supplementary file 2.

### Protein-protein interaction network analysis

PPI analysis displayed the signalling network and interactions among the DEPs (supplementary Fig. 2). The possible key regulators in the PPI network investigated by Cytoscape Hubba was shown in supplementary Table 1.

### Validation of proteins

**Fig. 4:**
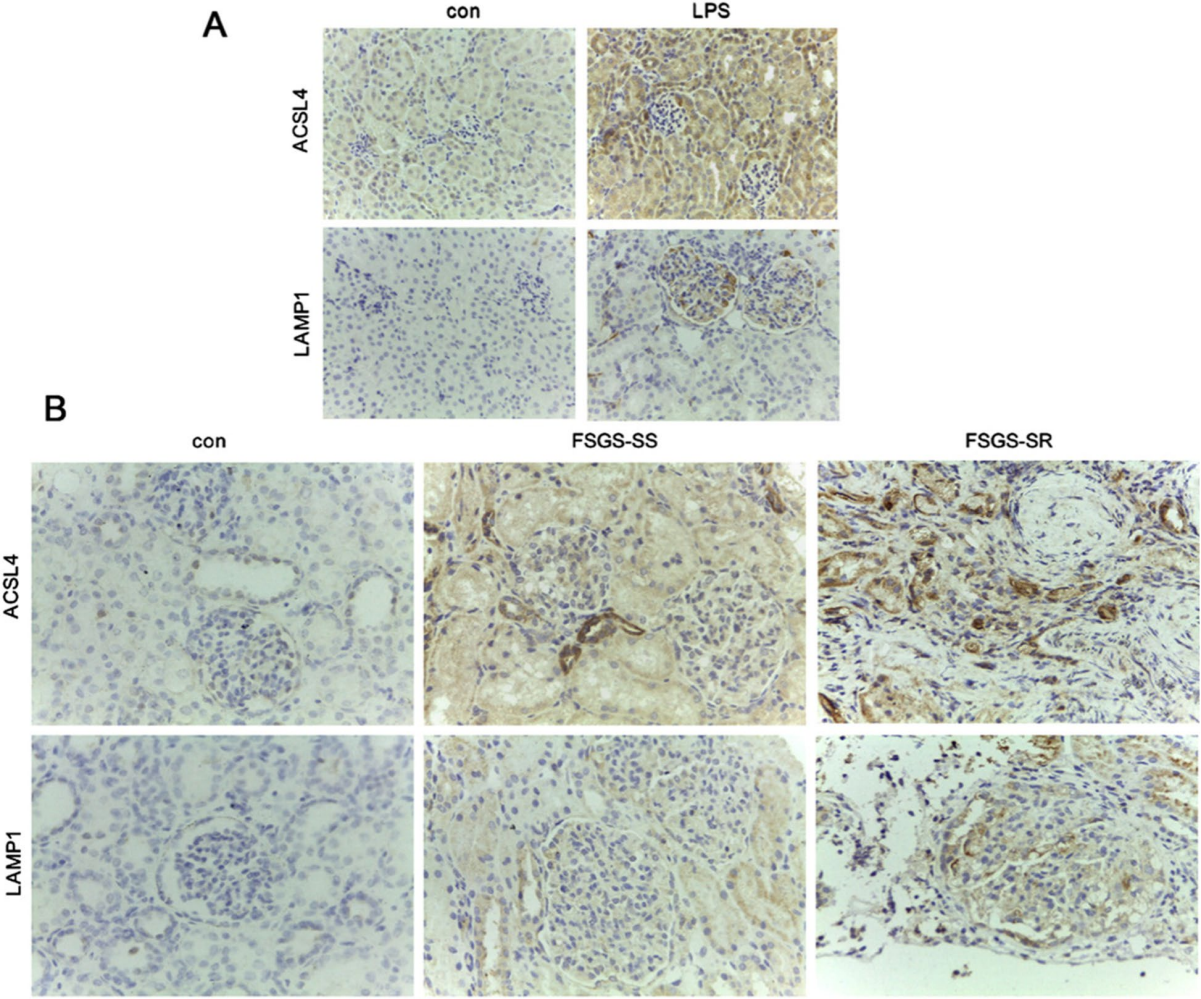
Validation analysis of ACSL4 and LAMP1. **A** Immunohistochemical stain for ACSL4 and LAMP1 of mouse kidney tissue after injury induced by LPS. **B** Immunohistochemical stain for ACSL4 and LAMP1 of the FSGS patients and controls. Original magnification, × 400

Among the top up-regulated proteins, the expression of long chain fatty acyl-CoA synthetase 4 (ACSL4) was further investigated in mouse and human kidney tissues to confirm the reliability of MS-based protein quantification. ACSL4 was significantly increased in kidney tissue especially in tubular epithelial cells in LPS mouse models (Fig. 4A). In addition, similar expression trend of ACSL4 was demonstrated in the kidney tissue from the FSGS-SR patient (Fig. 4B). In addition, lysosome associated membrane protein, LAMP1, was identified in the screening stage, consistent with expression trends that have been reported in the previous proteomic study of glomerular of FSGS with gene mutation [23]. As shown in Fig. 4A, B, LAMP1 significantly accumulated both in glomerulus of LPS mice models and FSGS-SR patient.

## Discussion

FSGS is the dominant cause of steroid-resistant nephrotic syndrome in children [24]. In recent years, the development of blood or urine-based proteomic biomarkers of SRNS and SSNS has attracted much interest [6–8]. One study on urinary-based proteome analysis of FSGS-SRNS and FSGS-SSNS identified some proteins, such as APOA1 (apolipoprotein A-1) and matrix-remodelling protein 8, as candidate urinary biomarkers of steroid sensitivity [7]. However, these proteins were not detected in the tissue samples in the present study which could be attributed to differences in the samples examined. A few reports exist in the literature on the global proteomic analysis of FFPE renal tissues in cases of lupus nephritis, rats with hypersensitivity and post-transplant kidney injury [25–27]. We performed this study on a small set of FFPE biopsies to uncover proteomic signatures associated with steroid effectiveness by using this proteomics platform. Furthermore, we revealed some novel proteins as well as other well-known proteins previously implicated in FSGS progression.

A series of studies have demonstrated the role of the complement system in the development of tubulointerstitial scarring in kidney diseases [28, 29]. In this study, we found that most of the up-regulated proteins are cytosolic and are primarily involved in physiological and pathological processes, including proteins associated with the regulation of complement activation processes. Several studies have demonstrated that complement inhibition effectively prevents progressive tubulointerstitial injuries in proteinuric renal disease, such as IgA nephropathy, anti-neutrophil cytoplasmic antibody-associated glomerulonephritis and atypical hemolytic uremic syndrome [22, 28, 30, 31]. These data suggested that if complement activation indeed contributes to glomerular injury, the detection of complement activation fragments allows for the possible identification and treatment of patients with an activated complement system. In view of the increased expression of the terminal pathway of complement-related proteins in the FSGS-SR group, complement pathway inhibitors such as membrane attack complex inhibitors could be used for steroid-resistant patients.

Validation experiments were conducted on two proteins, LAMP1 and ACSL4, which exhibited similar trends of expression in proteomics analysis, in order to examine the potential status as steroid-resistant candidates for FSGS. LAMP1 belongs to the family of lysosome-associated membrane proteins. Its high ranking in the PPI analysis and novelty made it stand out to us. Recently, a proteomic study of individual glomeruli from patients showed similar elevation of LAMP1 and indicated the dyregulation of proteolysis in FSGS [23]. In line with these results, our study also revealed a higher level of LAMP1 protein expression in FSGS-SR FFPE tissues than in FSGS-SS tissues. These results further indicated that the disruption of lysosome function leads to kidney injury and might play an important role in the steriod resistance of FSGS. Alternatively, forced expression of ACSL4 is observed in a variety of tumors, including hepatocellar carcinoma, colon adenocarcinoma, and aggressive breast cancer [32–34]. ACSL4 also regulates the expression of ATP-binding cassette (ABC) transporter associated with multi-drug resistance [35]. Conversely, reports have been rare regarding its function in renal disease. Recently, wang et.al found increased expression levels of ACSL4 in diabetic nephropathy mice, while ACSL4 inhibitor rosiglitazone could improve kidney function [36]. In this work, ACSL4 was elevated in FSGS-SR kidney tissue and was mainly expressed in tubular lesions. Whether the regulation of ABC transporter expression by ACSL4 is also involved in the mechanism of FSGS hormone resistance needs further study and ACSL4 may become the new molecular target for particular type of FSGS drug development.

In sum up, the results of our study offer valuable proteins and pathways to advance our understanding and management of FSGS. Several limitations and areas of potential improvements are highlighted for guiding future studies. Our research was a retrospective analysis with a small sample size and did not eliminate the effect of steroid or CNIs use before biopsy. Thus, we could not exclude the possibility that these treatments affected the detection of some potentially important proteins. Interesting insights into FSGS biology were still provided in this study, but a larger sample size with independent cohorts is necessary to confirm these findings. Despite such a small amount of kidney tissue, we were able to obtain substantial proteomic pattern differences, as well as pathway enrichments by this proteomic platform.

## Conclusions

In this study, the potential mechanism and candidates related to steroid resistance in children FSGS patients were identified. These proteomic variations may facilicate the identification of novel therapeutic targets for personalized care and a better prediction of patient outcomes.

## Supplementary Information

**Additional file 1.**

**Additional file 2.**

**Additional file 3.**

**Additional file 4: Supplementary Table 1.** Top 10 hub genes selected by Cytoscape Hubba. **Supplemental Fig. 1.** Enrichment GO function analysis of the upregulated proteins with signifificant difffferences between the groups. Only the leading terms of each group are presented, based on significance. A) GOCC terms analysis of upregulated proteins. B) GOMF analysis of upregulated proteins. GOCC, gene ontology term for cellular compoents; GOMF, gene ontology term for molecular function. **Supplemental Fig. 2.** Protein interaction network associated with FSGS derived from String online software analysis.

## Data Availability

The mass spectrometry proteomics data have been deposited to the ProteomeXchange Consortium (http://www.ebi.ac.uk/pride/archive/projects/PXD036164). All data generated during this study are included in this published article.

## Abbreviations

FSGS: Focal segmental glomerulosclerosis
SRNS: Steroid resistant nephrotic syndrome
FFPE: Formalin-fixed paraffin embedded
LC-MS/MS: Liquid chromatography tandem mass spectrometry
SS: Steroid-sensitive
KEGG: Kyoto Encyclopedia of Genes and Genome
GO: Gene Ontology
PPI: Protein-protein interaction
LAMP1: Lysosome associated membrane protein 1
ACSL4: Long chain fatty acyl-CoA synthetase 4
MCD: Minimal change disease
CNI: Calcineurin inhibitors
ESRD: End-stage renal disease
URPs: Up-regulated proteins
DEP: Differentially expressed protein groups
CC: Cell component
BP: Biological process
MF: Molecular function
ABC: ATP-binding cassette
