# Supplementary material for: Comparative proteomic analysis of children FSGS FFPE tissues
Source: BMC Pediatr. 2022 Dec 12;22:707. doi: 10.1186/s12887-022-03764-7 (PMC9743561; doi:10.1186/s12887-022-03764-7)

Supplementary Table 1. Top 10 hub genes selected by Cytoscape Hubba.

| Protein name | UniProt number | Degree score | Log2  (Fold change) | P value |
| --- | --- | --- | --- | --- |
| STAT3 | P40763 | 21 | 1.078 | 0.031 |
| RAB1A | P62820 | 19 | 3.929 | 0.009 |
| RAB8A | P61006 | 18 | 1.285 | 0.005 |
| VAMP7 | P51809 | 15 | 1.897 | 0.045 |
| F2 | P00734 | 15 | 1.296 | 0.028 |
| STXBP1 | P61764 | 14 | 10.526 | 0.025 |
| TCEB1 | Q15369 | 13 | 14.350 | 0.005 |
| VTN | P04004 | 13 | 1.453 | 0.001 |
| LAMP1 | P11279 | 13 | 1.042 | 0.019 |
| VPS52 | Q8N1B4 | 11 | 10.042 | 0.029 |

**Supplementary figure legends**

**Supplemental Fig. 1 Enrichment GO function analysis of the upregulated proteins with signifificant difffferences between the groups.** Only the leading terms of each group are presented, based on significance. A) GOCC terms analysis of upregulated proteins. B) GOMF analysis of upregulated proteins. GOCC, gene ontology term for cellular compoents; GOMF, gene ontology term for molecular function.

**Supplemental Fig. 2 Protein interaction network associated with FSGS derived from String online software analysis.**

**Fig. 1**


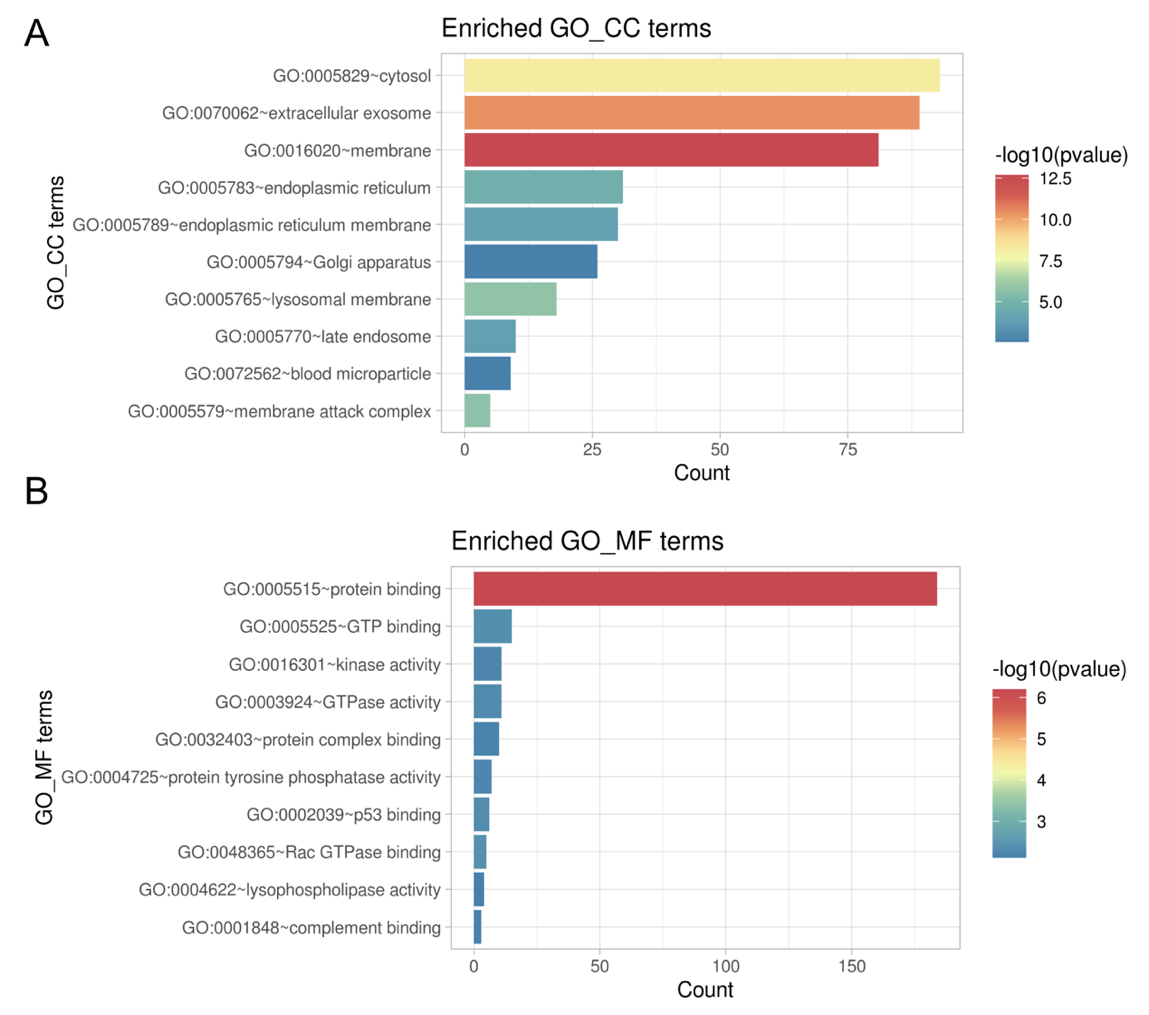


**Fig. 2**


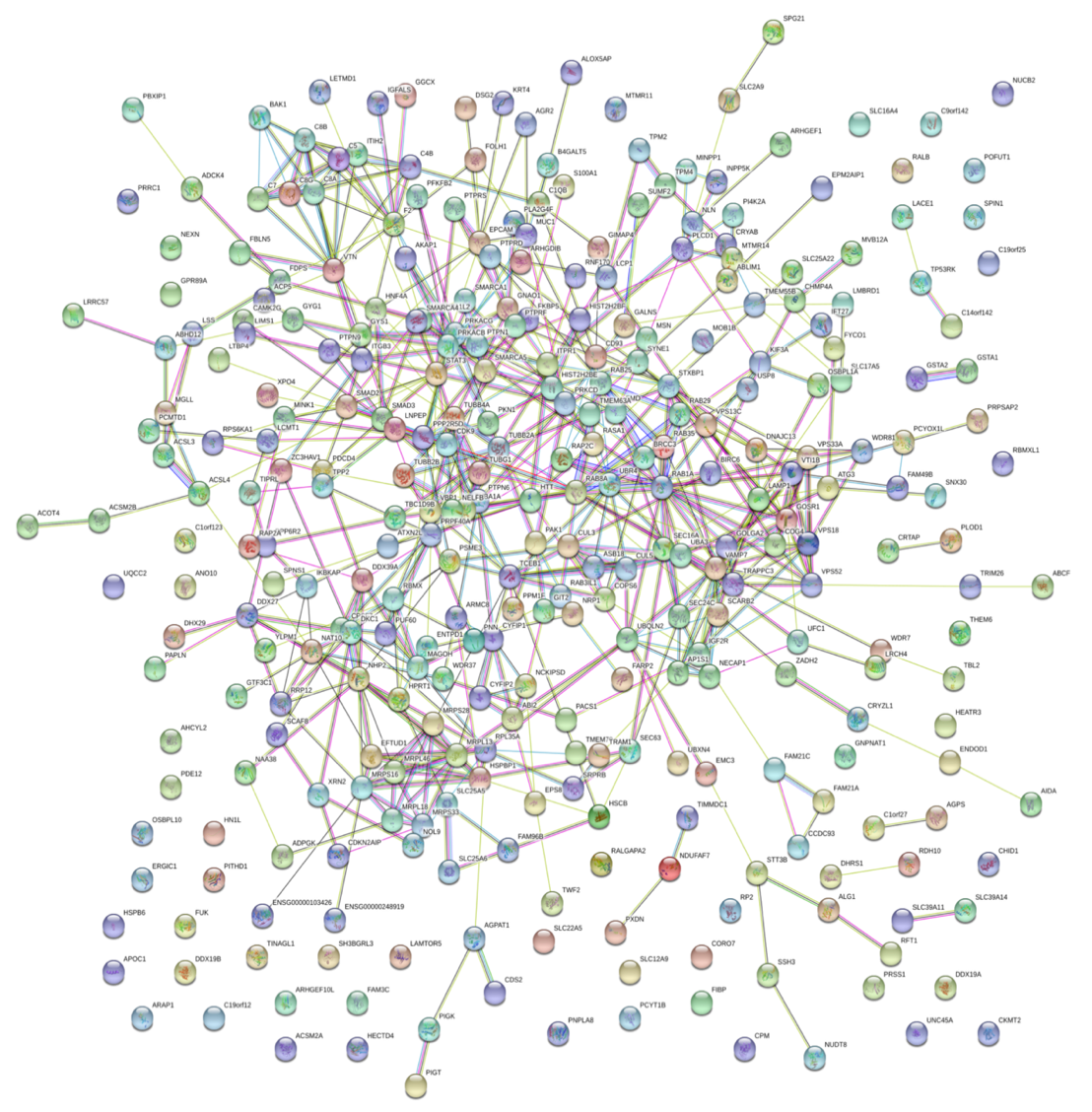

Supplement: Supplementary file 4 — Additional file 4: Supplementary Table 1. Top 10 hub genes selected by Cytoscape Hubba. Supplemental Fig. 1. Enrichment GO function analysis of the upregulated proteins with signifificant difffferences between the groups. Only the leading terms of each group are presented, based on significance. A) GOCC terms analysis of upregulated proteins. B) GOMF analysis of upregulated proteins. GOCC, gene ontology term for cellular compoents; GOMF, gene ontology term for molecular function. Supplemental Fig. 2. Protein interaction network associated with FSGS derived from String online software analysis. [file 12887_2022_3764_MOESM4_ESM.docx]
